# Medial and Lateral Meniscus Posterior Root Tears with an Intact Anterior Cruciate Ligament

**DOI:** 10.1155/2020/8842167

**Published:** 2020-07-18

**Authors:** Yuki Okazaki, Takayuki Furumatsu, Yuya Kodama, Yoshinori Matsumoto, Motoki Takahashi, Toshifumi Ozaki

**Affiliations:** ^1^Department of Orthopaedic Surgery, Okayama University Hospital, 2-5-1 Shikata-cho, Kita-ku, Okayama 700-8558, Japan; ^2^Department of Orthopaedic Surgery, Iwakuni Medical Center, 1-1-1 Atagomachi, Iwakuni, Yamaguchi 740-8510, Japan; ^3^Department of Orthopaedic Surgery, Takinomiya General Hospital, 486 Takinomiya, Ayakawa-cho, Ayauta-gun, Kagawa 761-2305, Japan

## Abstract

**Background:**

Medial meniscus (MM) posterior root tear (PRT) is often caused by meniscal degeneration, whereas lateral meniscus (LM) PRT is mainly caused by trauma, especially trauma associated with anterior cruciate ligament (ACL) injuries. Although there are a few reports on PRTs of both menisci with an ACL injury, to our knowledge, there is no report on those with an intact ACL. Thus, the purpose of this study was to describe a rare case of both meniscal PRTs with an intact ACL. *Case Presentation*. A 67-year-old woman complained of right knee pain during weeding in a deep knee flexion position. At presentation, three days after the injury, physical examination revealed signs of meniscal injury without ACL rupture. Magnetic resonance imaging showed PRTs of both menisci and damaged cartilage, especially on the medial femoral condyle and lateral tibial plateau. MM was sutured using the FasT-Fix dependent modified Mason-Allen suture technique, and LM by a single simple stitch using the Knee Scorpion suture passer. Referring to previous cadaveric studies, transtibial pullout repair using a single tibial tunnel for MM fixation was performed. The stability of the repaired menisci was checked by probing during second-look arthroscopy at one year after the primary surgery, and no meniscal signs and symptoms were present at the last follow-up one year after the surgery.

**Conclusions:**

This rare case showed PRTs of both menisci with an intact ACL. We speculated that, in this case, both roots tore because of the degenerative menisci. A good clinical outcome was achieved after single-transtibial pullout repair. This technique may be an effective surgical approach for PRTs of both menisci.

## 1. Introduction

Posterior root attachments of the menisci play an important role in the maintenance of the articular cartilage of the knee. Both radial tear and posterior horn avulsion result in meniscal dysfunction of load-bearing structures, with increasing local contact pressure and premature onset of knee arthritis [1]. Lateral meniscus (LM) posterior root tear (PRT) is mainly caused by trauma and mostly associated with anterior cruciate ligament (ACL) injury, whereas medial meniscus (MM) PRT is usually caused by chronic degenerative meniscal disease. Although there are a few reports on PRTs of both menisci with an ACL injury [2, 3], to our knowledge, there is no report on those without ACL injury. We considered a degenerative origin for both the medial and lateral menisci. Here, we describe a rare case of both meniscal PRTs with an intact ACL in a middle-aged woman.

## 2. Case Presentation

**Figure 1:**
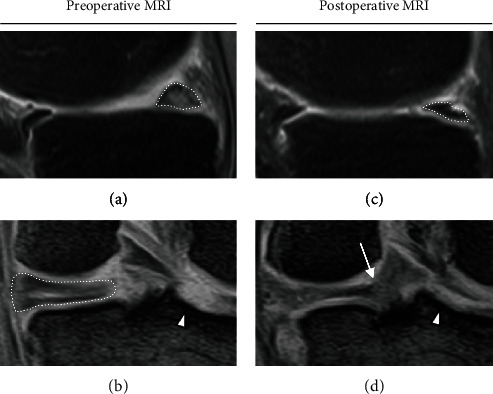
Magnetic resonance imaging. (a) Ghost sign (dotted area) on sagittal view. (b) Giraffe neck sign (dotted area) and highly damaged articular cartilage on the lateral tibial plateau beside the lateral tibial eminence (arrowhead) on coronal view. (c) Repaired medial meniscus (MM) root (dotted area). (d) Continuity of the repaired MM root (arrow) and improved articular cartilage (arrowhead).

**Figure 2:**
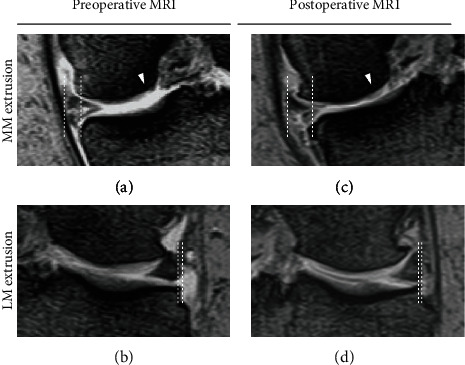
Magnetic resonance imaging—coronal view. (a) Preoperative medial meniscus extrusion (MME) (4.3 mm) and highly damaged articular cartilage on the medial femoral condyle (arrowhead). (b) Preoperative lateral meniscus extrusion (LME) (1.2 mm). (c) Postoperative MME (7.0 mm) and improved articular cartilage (arrowhead). (d) Postoperative LME (0.9 mm).

**Figure 3:**
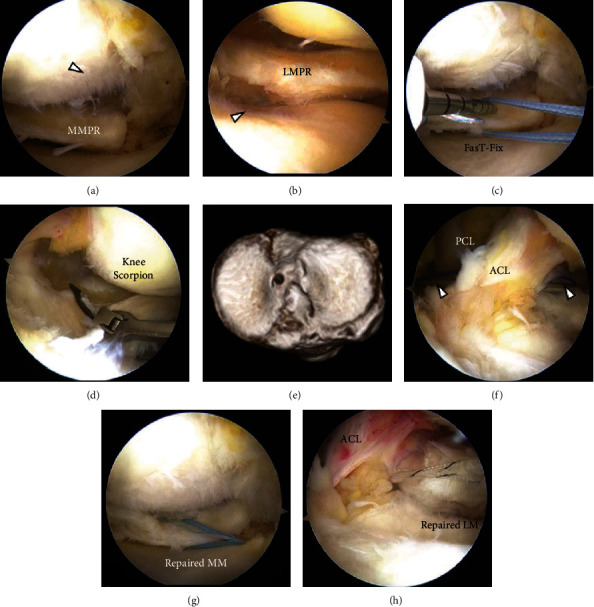
Arthroscopic findings and three-dimensional computed tomography. (a) Medial meniscus posterior root tear and highly damaged articular surface on the medial femoral condyle (arrowhead). (b) Lateral meniscus posterior root tear and highly damaged articular surface on the lateral tibial plateau (arrowhead). (c) FasT-Fix (Smith & Nephew, Andover, MA, USA) dependent modified Mason-Allen suture technique. (d) A simple stitch suture using the Knee Scorpion suture passer (Arthrex, Naples, FL, USA). (e) Position of the tibial tunnel. (f) Suture is passed between the ACL and the PCL using a suture retriever (Smith & Nephew) (arrowhead). (g) Repaired MM. (h) Repaired LM. MM: medial meniscus. LM: lateral meniscus. ACL: anterior cruciate ligament. PCL: posterior cruciate ligament. MMPR: medial meniscus posterior root. LMPR: lateral meniscus posterior root.

A 67-year-old woman complained of right knee pain during weeding in the deep knee flexion position. At presentation, three days after the injury, physical examination revealed signs of meniscal injury but not those of ACL rupture. Full flexion was painful and restricted by knee swelling. Manual knee laxity tests, including the Lachman and anterior drawer test, were negative, whereas the McMurray test was positive. The femoral tibial angle and the Kellgren-Lawrence grade on the preoperative radiograph were 171° and 1, respectively. Magnetic resonance imaging (MRI) showed high medial tibial slope (MTS) (6.5°) and relatively low lateral tibial slope (LTS) (4.1°), PRTs of both menisci (Figures 1 and 2) [4, 5], and damaged cartilage, especially on the medial femoral condyle (MFC) and lateral tibial plateau (LTP) (Figures 1(b) and 2(a)), which were considered to be caused by MMPRT and LMPRT, respectively. Arthroscopic evaluation under general anesthesia revealed these lesions, and no other intraarticular lesions, including meniscofemoral ligament tear, were detected (Figures 3(a) and 3(b)).

**Figure 4:**
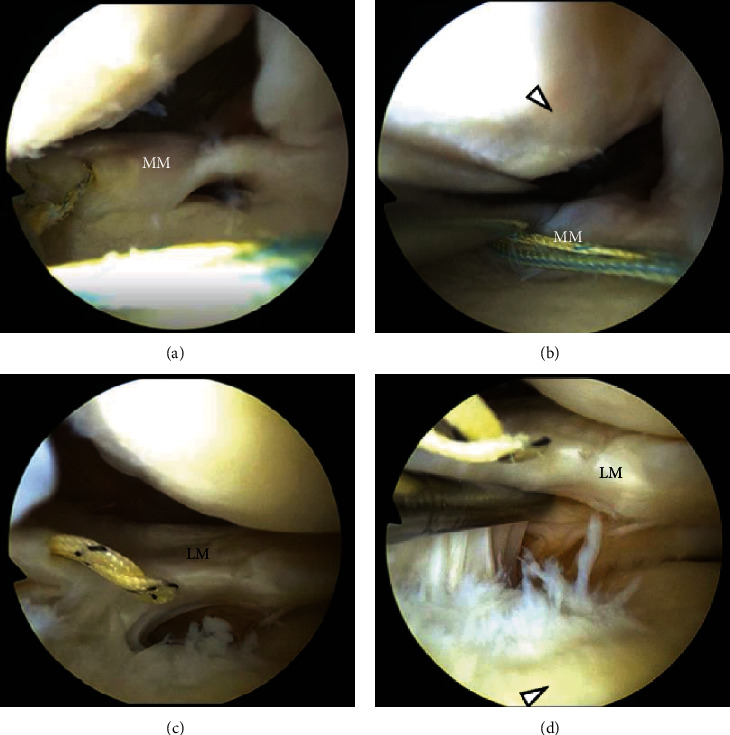
Second-look arthroscopic findings. (a) Repaired medial meniscus (MM) attached to the tibial footprint. (b) Improved articular cartilage on the medial femoral condyle (arrowhead) and stable MM checked by probing under the 60° knee-flexed position. (c) Repaired LM attached to the tibial footprint. (d) Improved articular cartilage on the lateral tibial plateau (arrowhead) and enough synovial coverage around the LM footprint checked by probing.

The MM was sutured by FasT-Fix (Smith & Nephew, Andover, MA, USA) dependent modified Mason-Allen suture technique (Figure 3(c)) [6], whereas the LM was sutured by a single simple stitch using the Knee Scorpion suture passer (Arthrex, Naples, FL, USA) and #2 FiberWire (Arthrex) (Figure 3(d)). Transtibial pullout repair was performed using a single tibial tunnel for MM fixation. Bone tunnel preparation was performed using an MMPRT guide, as described previously [6] (Figure 3(e)). Both suture ends were pulled out through the tibial tunnel after the LM suture was passed between the ACL and the posterior cruciate ligament using a suture retriever (Smith & Nephew) (Figure 3(f)). Tibial fixation was performed under an initial tension of 20 N in the 45° knee-flexion position (Figures 3(g) and 3(h)). The postoperative rehabilitation program was the same as that after MMPRT pullout repair, as described previously [6]. The stability of the repaired menisci was checked by probing during second-look arthroscopy one year after the primary surgery (Figure 4). At the last follow-up one year after the surgery, no meniscal signs and symptoms were present. The McMurray test was negative, and the patient could easily perform “seiza.” The Kellgren-Lawrence grade on postoperative radiography was 2. Postoperative MRI showed good healing of both roots and articular cartilage (Figures 1(c), 1(d), 2(c), and 2(d)).

## 3. Discussion

This case showed PRTs of both menisci with an intact ACL, although the injury patterns for PRTs of both menisci are usually distinct. Generally, MM degeneration precedes LM degeneration; however, in our case, both menisci degenerated and tore simultaneously. Highly damaged articular cartilage lesions on MFC and LTP were identified on MRI and on primary arthroscopic surgery, although they were performed soon after injury, at two weeks and two months, respectively. We consider that the patient had a relatively low LTS and high MTS. The average LTS was reported to be about 5.0° [7], but LTS in the present case was 4.1°, which might have caused an abnormally flat LM posterior horn, resulting in the degenerative disorder [8]. Furthermore, MTS in the present case was 6.5°, although the mean MTS was reported to be about 4.8° [7] and 7.2 especially in MMPRT knee [9]. The tibial slope in the present case might result in PRTs of both menisci and cartilage degeneration in the medial tibia and parts of the lateral compartment [10].

The sequela of MMPRT left *in situ* or misdiagnosed is functionally equivalent to total meniscectomy, and pullout repair is recommended for MMPRT. There were a few reports of conservative treatment of LMPRT with ACL injury. However, there are many studies reporting negative outcomes with LMPRT left *in situ*. Following LMPRT, the peak tibiofemoral contact pressure increases from 2.8 to 4.2 MPa, but the pressure returns to normal after pullout repair via a transtibial tunnel [3]. Furthermore, when root avulsion occurs, the meniscofemoral ligament probably inhibits spontaneous healing because of continuous traction by the ligament during knee movements [2]. Thus, we speculate that spontaneous healing after LMPRT with an intact ACL is less probable, and pullout repair is suitable for restoring the function of original LM.

Forkel et al. reported an LMPRT pullout repair technique combined with arthroscopic ACL reconstruction. They reported using cadavers to compare anatomic fixation with fixation through the tibial ACL tunnel and showed no significant difference in the mean pressure between the two techniques; comparison of ACL tunnel fixation with the intact state also showed no significant difference [11], although the tibial ACL tunnel is anterior to the medial tibial eminence (MTE) apex. In fact, the posteromedial crus from the LM posterior horn runs between ACL and PCL and attaches to the posterior aspect of MTE, whereas the LM posterior root attachment center was 4.2 mm medial and 1.5 mm posterior from the lateral tibial eminence apex [12, 13]. Based on these studies, we considered that bone tunnel created for the MM was more suitable than a bone tunnel created at the LM posterior root attachment; further, this technique was minimally invasive and did not require an additional bone tunnel. Thus, we created the bone tunnel toward the original MM root attachment and also used this tunnel for LM fixation, as this was reasonable for LM posterior root attachment. In this rare case of both meniscal PRTs with an intact ACL, LM extrusion on MRI showed an improvement, and clinical evaluation after pullout repair using a single transtibial tunnel confirmed good healing. This technique may be an effective surgical approach for PRTs of both menisci.

## Data Availability

Data is available when asked.
